# Genome-wide Characterization of the *MBF1* Gene Family and Its Expression Pattern in Different Tissues and Under Stresses in *Medicago truncatula* and *Medicago sativa*

**DOI:** 10.3390/ijms26020455

**Published:** 2025-01-08

**Authors:** Yiran Zhao, Hao Wang, Rongfeng Liu, Kunlong Su, Guofeng Yang

**Affiliations:** College of Grassland Science, Qingdao Agricultural University, Qingdao 266109, China; zhaoyiran@qau.edu.cn (Y.Z.); w15253589581@163.com (H.W.); liu15847888804@163.com (R.L.)

**Keywords:** *Medicago truncatula*, *Medicago sativa*, *MBF1* genes, abiotic stresses, expression profiling

## Abstract

Multiprotein bridging factor 1 (MBF1) is a transcription factor family playing crucial roles in plant development and stress responses. In this study, we analyzed MBF1 genes in *Medicago truncatula* and *Medicago sativa* under abiotic stresses, revealing evolutionary patterns and functional differences. Four *MBF1* genes were identified in *M. truncatula* and two in *M. sativa*, with conserved MBF1 and HTH domains, similar exon/intron structures, and stress-related *cis*-elements in their promoters. Subcellular localization showed that MtMBF1a.1 is predominantly localized in the nucleus, while MtMBF1a.2, MtMBF1b, MtMBF1c, and MsMBF1a localize to both the nucleus and cytoplasm. In contrast, MsMBF1c is exclusively localized in the cytoplasm. An expression analysis revealed distinct stress responses: salt stress-induced *MtMBF1b* and *MtMBF1c* expression but repressed *MsMBF1a* and *MsMBF1c*. In contrast, PEG stress did not affect *M. truncatula MBF1* genes but repressed both *M. sativa MBF1* genes. These findings provide insights into MBF1-mediated stress adaptation and inform strategies for the molecular breeding of stress-tolerant alfalfa.

## 1. Introduction

Environmental stresses, including drought, salinity, and extreme temperatures, significantly impact plant growth and development. These stresses often lead to physiological and morphological changes, and in severe cases, even plant mortality [1]. To survive under such challenging conditions, plants have developed intricate and highly efficient information transfer systems [2]. Central to these systems are stress-inducible transcription factors (TFs), which allow plants to perceive and respond to environmental signals. Important transcription factors, such as basic leucine zipper (bZIP), Myeloblastosis (MYB), basic Helix–Loop–Helix (bHLH), WRKY [3,4], ethylene responsive factor (ERF) [5], NAC, MBF1, and many others, are then activated to mediate appropriate responses to environmental stress [6]. Among these, MBF1 serves as a critical transcriptional coactivator, essential for modulating gene expression and enabling plants to adapt to various environmental stresses [7]. The MBF1 family of proteins is characterized by a flexible N-terminal region and a highly conserved helix–turn–helix (HTH) domain located in the C-terminal region [7]. These features play a crucial role in facilitating interactions with activated proteins, enabling MBF1 to function as an effective transcription co-factor. This flexibility is essential for adapting to various protein partners and ensuring the proper formation of functional complexes. Functionally, the N-terminus of MBF1 facilitates binding to activated proteins, while the C-terminus plays a crucial role in protein folding and dimer formation [7]. Acting as a non-DNA binding transcription co-factor, MBF1 serves as a general factor for the TATA-box binding protein (TBP), which is indispensable for transcription initiation. To execute these diverse functions, MBF1 proteins exhibit dynamic localization, shuttling between the cytoplasm and nucleus [8]. Studies in yeast have revealed that MBF1 interacts directly with the members of the bZIP family of transcription factors, specifically GCN4, forming a GCN4-MBF1-TBP complex [9]. This intricate complex underscores the pivotal role of MBF1 in regulating transcription and highlights its involvement in enhancing a plant’s tolerance to both biotic and abiotic stress. The genus *Arabidopsis* contains three *MBF1* genes, which are broadly expressed across the plant body, including roots, stems, leaves, flowers, fruits, seeds, and anther organs [10]. Despite this widespread expression, studies have revealed varying expression abundances among the three *MBF1* genes during different stages of plant development [11,12].

Functional studies in *Arabidopsis* have highlighted the diverse roles of these genes. For example, the overexpression of *AtMBF1a* has been shown to result in glucose hypersensitivity, which enhances tolerance to both salt stress and pathogen infection [13]. Similarly, *AtMBF1c* overexpression not only increased seed yield in *Arabidopsis* and *Glycine max* [14,15], but also led to rapid accumulation and interaction with trehalose-phosphate synthase under heat stress [15]. The functional diversity of *MBF1* genes is also evident in other species. In grapes (*Vitis vinifera*), the overexpression of *VvMBF1* improved drought tolerance in transgenic *Arabidopsis thaliana* [16]. In rice (*Oryza sativa*), *TaMBF1c* overexpression conferred heat tolerance in response to high-temperature stress [17]. Conversely, in pepper (*Capsicum annuum*), the *MBF1* gene appears to act as a negative regulator of cold stress responses [10].

The perennial legume alfalfa (*Medicago sativa*) is an important crop globally. In terms of livestock feed, alfalfa is rich in protein, amino acids, vitamins, and minerals. These nutrients are essential for improving milk production, meat quality, and the overall health of livestock such as dairy cows, beef cattle, and sheep [18]. In terms of soil health, alfalfa, as a legume, has the ability to fix nitrogen. The rhizobia symbiotic with its roots can fix nitrogen in the air and convert it into ammonia nitrogen that can be used by plants, thereby increasing the nitrogen content in the soil. This process not only reduces the use of chemical fertilizers and reduces agricultural production costs, but also helps improve soil structure and increase soil fertility and water retention capacity [19]. The growth of alfalfa is severely limited by various unfavorable environmental factors [20], highlighting the urgent need to identify key genes that can enhance its tolerance to environmental stresses and improve yields [21]. While tetraploid alfalfa has a complex genome and is particularly vulnerable to environmental stresses, it shares an evolutionary relationship with *Medicago truncatula*, a legume diploid species extensively studied as a model for plant development and stress responses. Specifically, we note that the tetraploid genome of alfalfa is complex and includes multiple homologous gene copies, which can both enhance genetic redundancy and complicate functional gene analyses [22]. This genomic redundancy offers a buffering capacity against environmental stresses, potentially aiding in stress resilience. This connection suggests that a comparative analysis of *M. truncatula* and *M. sativa* could offer valuable insights into the genetic advantages and genomic resources of alfalfa. Although the *MBF1* genes in several *Medicago* species have been documented [8,11,16,17], and their roles in plant growth and development are well established, little is known about the functional role of these genes specifically in *Medicago* plants. In this study, we identified *MBF1* genes from *M. truncatula* and *M. sativa* and analyzed their multiple sequence alignments, phylogenetic relationships, gene structures, protein motifs, and *cis*-acting elements. We also analyzed the expression profiles of the *MBF1* genes of *M. truncatula* and *M. sativa* in response to salt, drought, cold and heat stresses. Our comparative investigation of the *MBF1* genes of the two *Medicago* species will facilitate the functional characterization of individual *MBF1* genes that respond to environmental stresses and developmental signals in *Medicago* plants. Overall, our study aims to provide an empirical basis for future work on the role of *MBF1* genes in mediating abiotic stress responses in plants.

## 2. Results

### 2.1. Identification of MBF1 Genes in the M. truncatula and M. sativa Genomes

Based on domain confirmation and a homology search, we identified a total of four putative *MBF1* genes (*MtMBF1a.1*, *MtMBF1a.2*, *MtMBF1b*, and *MtMBF1c*) in *M. truncatula* and two putative *MBF1* genes (*MsMBF1a* and *MsMBF1c*) in *M. sativa* (Table 1). Tomato (*Solanum lycopersicum*) was used as a reference due to its well-characterized genome and evolutionary relevance to *Medicago*, facilitating comparative analysis of *MBF1* gene structure. The analyses of their molecular properties showed that most MBF1 proteins of the two *Medicago* species were between 110 aa and 150 aa, making them similar in length to their MBF1 counterparts in tomato [23], with the exceptions of MtMBF1c and MsMBF1a, which were shorter in length, at 56 aa and 74 aa, respectively. The predicted pI values ranged from 8.6 (MtMBF1b) to 10.24 (MsMBF1c), while the predicted molecular weights ranged from 6.18 kDa (MtMBF1c) to 15.6 kDa (MtMBF1a.1). The corresponding homologous *MBF1* genes of *M. sativa* and *M. truncatula* were identified in parallel by sequence alignment.

### 2.2. Multiple Sequence Alignment, Phylogenetic and Gene Structure Analyses of MBF1 Genes in Medicago

*Arabidopsis* and *Oryza sativa* were chosen as model species for the comparison because they represent dicots and monocots, respectively, enabling the identification of the conserved and lineage-specific characteristics of *MBF1* genes. We compared 11 protein sequences of *Arabidopsis*, *Oryza sativa*, and *Medicago* by using the MUSCLE method. Sequence alignments of multiple amino acids between OsMBF1s, AtMBF1s, MtMBF1s, and MsMBF1s indicated that the sequences were highly conserved (Figure S1); all the MBFs contained the MBF1 domain in the N-terminus (MtMBF1b has an incomplete MBF domain) and the HTH domain in the C-terminus (except in MsMBF1a).

**Figure 1:**
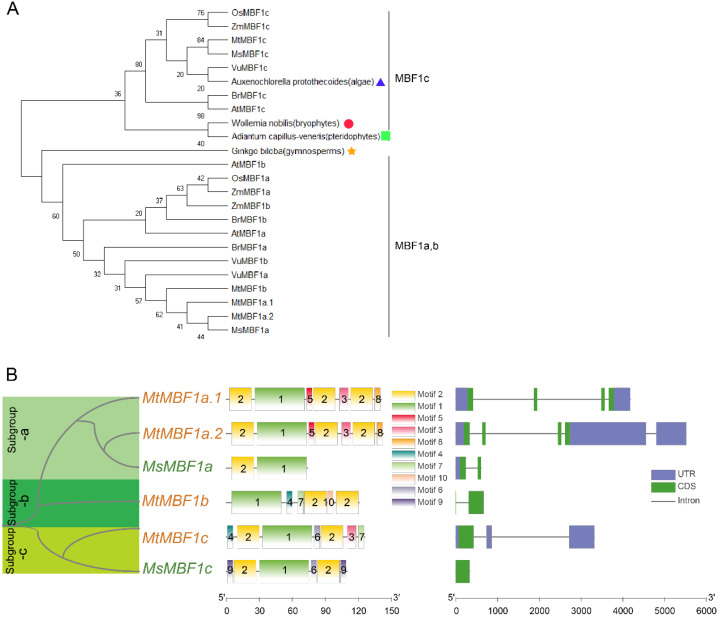
Phylogenetic relationships, motifs, and gene structures of *MBF1* genes. (**A**) Phylogenetic analysis of *MBF1* families across *Medicago*, *Arabidopsis*, *Oryza sativa*, *Zea mays*, *Vigna unguiculata*, *Brassica rapa*, algae, bryophytes, pteridophytes, and gymnosperms. Full-length protein sequences of MBF1s were constructed using MEGA-X based on the neighbor-joining (NJ) method with 1000 bootstraps. Blue triangles, red circles, green squares, and orange pentagrams represent algae, bryophytes, pteridophytes, and gymnosperms, respectively. (**B**) Phylogenetic relationships, motifs, and gene structures of the *MBF1* genes from *M. truncatula* and *M. sativa*. The motifs are indicated by different colored boxes with different numbers. Blue boxes indicate 5′- and 3′-untranslated regions; green boxes indicate exons; black lines indicate introns.

A sequence-based phylogenetic analysis among *M. truncatula*, *M. sativa*, *Arabidopsis*, *O. sativa*, *Z. mays*, *V. unguiculata*, and *B. rapa* showed that these MBF1 proteins were grouped into two distinct clades (ab, c) (Figure 1A). The MBF1 proteins from representative species of algae, bryophytes, ferns, and gymnosperms were selected to better visualize the evolutionary relationships of MBF1 across different taxa. Sporophytes, which have relatively simple organ structures, clustered with MBF1c. This suggested that MBF1c may be the ancestral member of the phylogeny. MBF1a and MBF1b displayed a close relationship (Figure 1A), suggesting that the two proteins may share similar functions. The phylogenetic analysis revealed that MBF1c is grouped in a distinct clade from MBF1a and MBF1b (Figure 1A), indicating potential functional divergence. Although MBF1c has been implicated in developmental processes, its role in stress response cannot be excluded and requires further study.

To understand the structural characteristics of *MBF1* genes in *Medicago*, we analyzed their motifs and exon/intron structures. Overall, the results were consistent with those of the phylogenetic analyses (Figure 1B). Specifically, ten conserved motifs were detected in the MBF1 proteins. Motifs 1 and 2 were detected in all the MBF1 proteins; motif 1 is an MBF1 domain and motif 2 is an HTH domain (Figure 1B). Given that MBF1 proteins in *Medicago* display similar patterns of motif distribution, these patterns may be used to predict or distinguish the functions of the different proteins.

We found that the exon numbers of the *MBF1* genes of the two *Medicago* species ranged between one and four, while their intron numbers ranged between zero and three (Figure 1B). Additionally, *MtMBF1a.2* and *MtMBF1c* contained many untranslated regions (UTR) sequences, which accounted for 55% and 46% of their gene sequences, respectively. In contrast, *MtMBF1b* and *MsMBF1c* did not contain UTR. Differences in exon or intron length were also observed between the orthologous members of the MBF1 family.

### 2.3. Analysis of Chromosome Location and Collinearity of MBF1 Genes

**Figure 2:**
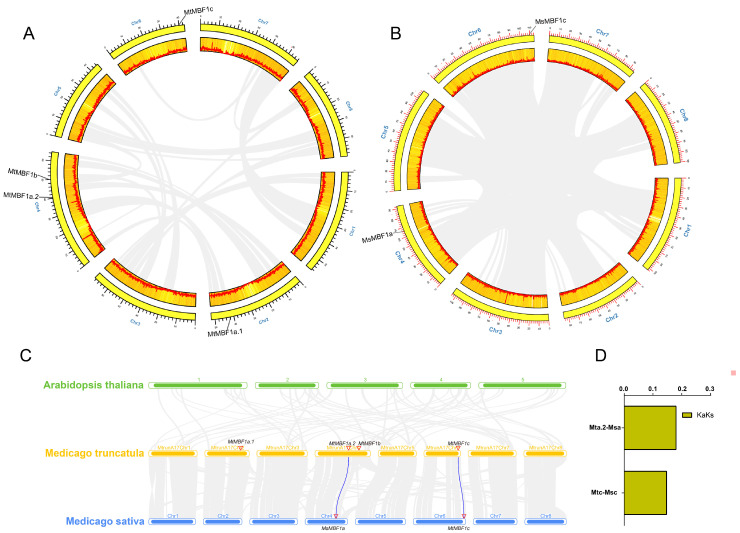
Chromosome distributions of *MBF1s* in *M. truncatula* and *M. sativa*. The chromosomal location and interchromosomal relationship of *M. truncatula* (**A**) and *M. sativa* (**B**). (**C**) Synteny analysis of *MBF1* genes between *Arabidopsis*, *M. truncatula*, and *M. sativa*. The gray lines in the background indicate collinear blocks between *M. truncatula*, and *M. sativa*/*Arabidopsis*, while the blue lines highlight syntenic *MBF1* gene pairs. (**D**) The Ka/Ks values of *MBF1* gene pairs for *Mt-Ms*.

The chromosome location data of each *MBF1* gene was downloaded from the Phytozome database (https://escholarship.org/uc/item/22k9d6k9) (accessed on 12 October 2022). Using the data, we analyzed the *MBF1* genes and mapped them onto their corresponding chromosomes using TBtools (Figure 2A,B). We found that the *MBF1* genes were distributed unevenly in both *M. truncatula* and *M. sativa*. Specifically, *MtMBF1* was distributed on chromosomes 2, 4, and 6, while the two other genes (*MtMBF1a.2* and *MtMBF1b*) were distributed on chromosome 4 (Figure 2A). *MsMBF1a* and *MsMBF1c* were distributed on chromosomes 4 and 6, respectively. (Figure 2B). In addition, there were neither segmental duplication nor tandem duplication events detected in both *M. truncatula* and *M. sativa*.

Gene synteny analysis plays a critical role in understanding the evolutionary relationships between species. In the context of plant genomics, synteny refers to the conservation of gene order across different genomes, which can help identify shared ancestry and evolutionary patterns. We constructed three comparative syntenic maps for *M. sativa*, *M. truncatula*, and their associations with representative *Arabidopsis* species to illustrate the evolutionary relationships of the *MBF1* genes family (Figure 2C). We found that two pairs of genes were covalently related between *M. sativa* and *M. truncatula*. Meanwhile, *MtMBF1a.2* was collinear with *MsMBF1a*, and *MtMBF1c* was collinear with *MsMBF1c*. To gain a better understanding of the evolutionary selection pressures driving the formation of the *MBF1* gene family, we analyzed non-synonymous/synonymous substitution (Ka/Ks) values of *MBF1* gene pairs between *M. sativa* and *M. truncatula*. Generally, Ka/Ks > 1, Ka/Ks = 1, and Ka/Ks < 1 indicate positive selection, neutral evolution, and purifying selection, respectively [24]. We found that the ratios of two (*MtMBF1a.2*-*MsMBF1a* and *MtMBF1c*-*MsMBF1c*) homologous pairs were smaller than 0.2 (Figure 2D), suggesting that these genes had undergone purifying selection after segmental and whole genome duplications.

### 2.4. Analysis of Cis-Acting Element of MBF1 Genes

**Figure 3:**
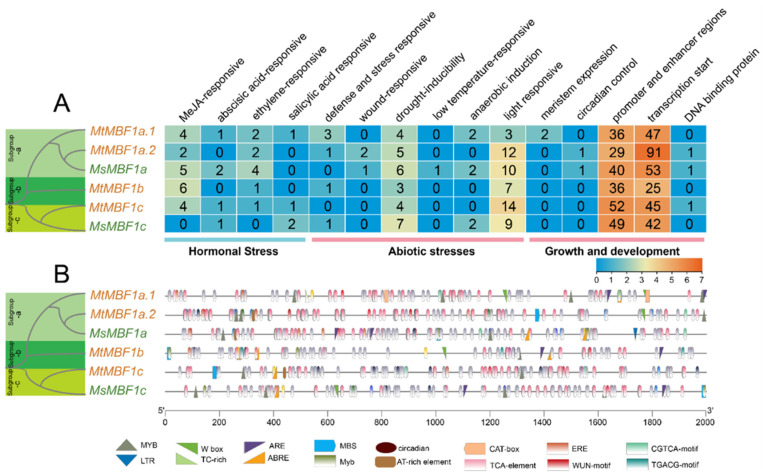
Putative *cis*-elements and transcription factor binding sites in the promoter regions of *MBF1* genes from *M. truncatula* and *M. sativa*. (**A**) The color and number of the grid indicate the numbers of different *cis*-acting elements in these *MBF1* genes. (**B**) The colored block represent different types of *cis*-acting elements and their locations in each *MBF1* gene.

To further determine the molecular functions and expression patterns of the *MBF1* family, we focused on the *cis*-acting elements (1500 bp upstream of the ATG start codon) related to plant hormones, abiotic stress, growth, and development (Figure 3). In phytohormone pathways, most of the *MBF1* genes were found to have more *cis*-elements involved in methyl jasmonate (MeJA) and ethylene responsiveness (Figure 3). In addition, a series of *cis*-acting elements were found to be associated with abiotic stress. All the *MBF1* genes were found to be primarily involved in the plant’s responsivity to drought and light. Given that their *cis*-elements were related to hormone and abiotic stress functions, it was inferred that the *MBF1* gene family had a strong response to external environmental stimuli. In addition, the *MBF1* gene family contains many promoter enhancement regions and transcription initiation elements, which critically determine the rapid response of plants to external stimuli. Notably, a unique *cis*-element associated with circadian control was obtained in *MtMBF1a.2* and *MsMBF1a*. This suggested that *MBF1* genes may play a role in regulating the circadian rhythms of plants.

### 2.5. Analysis of Subcellular Localization of MBF1s

**Figure 4:**
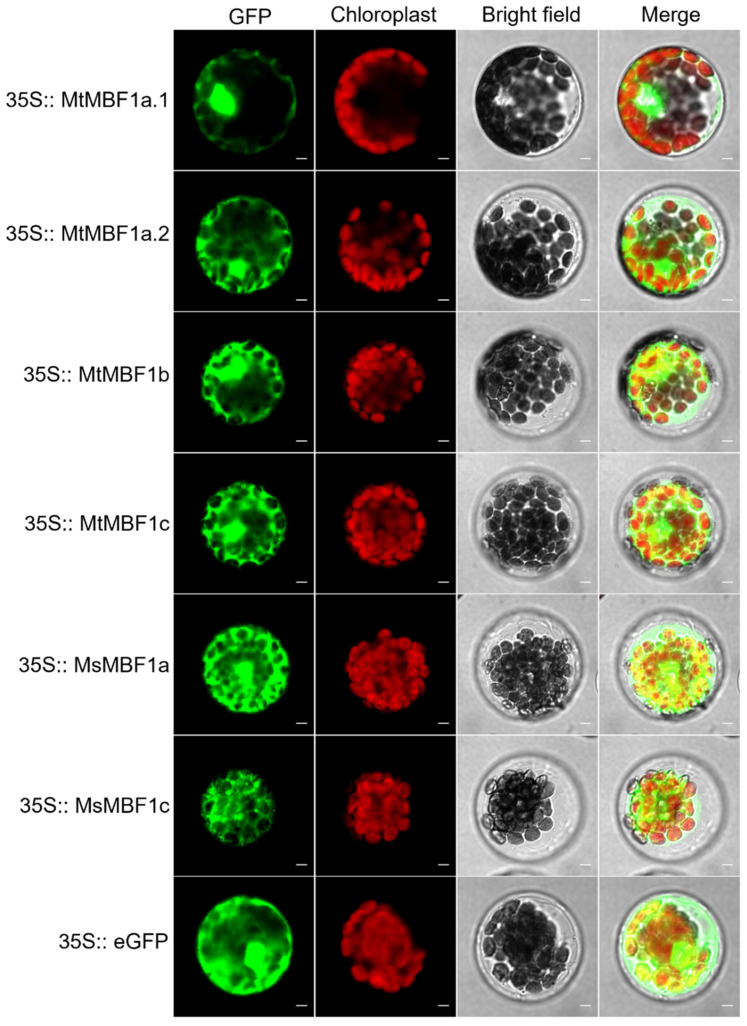
Subcellular localization assay of MBF1s. The coding region of *MBF1*s was cloned into the pCAMBIA1300-35S-eGFP vector. This construct was expressed transiently in *Arabidopsis* mesophyll protoplasts using the PEG-mediated method. eGFP, enhanced GFP. Scale bar = 10 μm.

We conducted the subcellular localization assay of the six MBF1 proteins using the transient expression of MBF1-eGFP fusions in *Arabidopsis* mesophyll protoplasts. The results showed that MtMBF1a.1 was predominantly localized in the nucleus, whereas MtMBF1a.2, MtMBF1b, MtMBF1c, and MsMBF1a displayed strong fluorescence in both the nucleus and cytoplasm. In contrast, MsMBF1c fluorescence was detected only in the cytoplasm (Figure 4). These findings suggest that MtMBF1a.1 may primarily function in the nucleus, distinguishing it from other MBF1 proteins.

### 2.6. Determining MBF1s Expression Patterns in Different Tissue Sites by qRT-PCR

**Figure 5:**
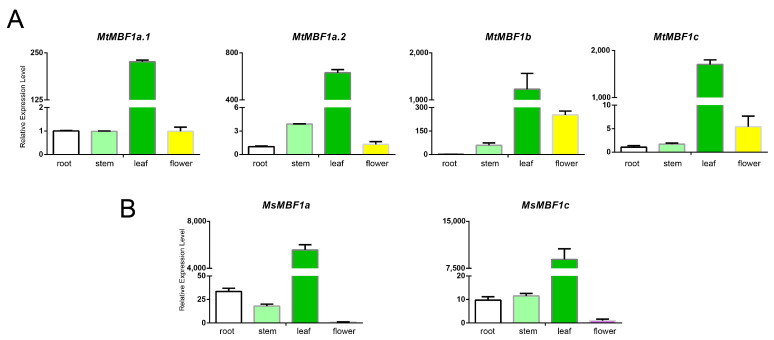
Determining *MBF1* expression patterns in different tissue sites by qRT-PCR. (**A**) Expression levels of *MtMBF1* genes in various tissues verified by qRT-PCR. (**B**) Expression levels of *MsMBF1* genes in various tissues verified by qRT-PCR.

To understand the potential functions of *MBF1* genes in *Medicago* plants, their expression patterns were examined via qRT-PCR in different organs, including the roots, stems, leaves, and flowers (Figure 5A,B). All four *MBF1* genes were detected in these tissues (Figure 5A,B). Remarkably, the expression levels of all the genes in the leaves were significantly higher than those contained in the other tissues. Notably, *MBF1* gene expression was significantly higher in *M. sativa* than in *M. truncatula* (*MsMBF1c* up to 8000 in leaves and *MtMBF1c* up to 1800 in leaves) (Figure 5A, B). Meanwhile, the expression of the *MBF1* gene was the lowest in the roots of *M. truncatula* and lowest in the flowers of *M. sativa*.

### 2.7. Expression Patterns and Correlation Analysis of MBF1 Genes Under Abiotic Stress

**Figure 6:**
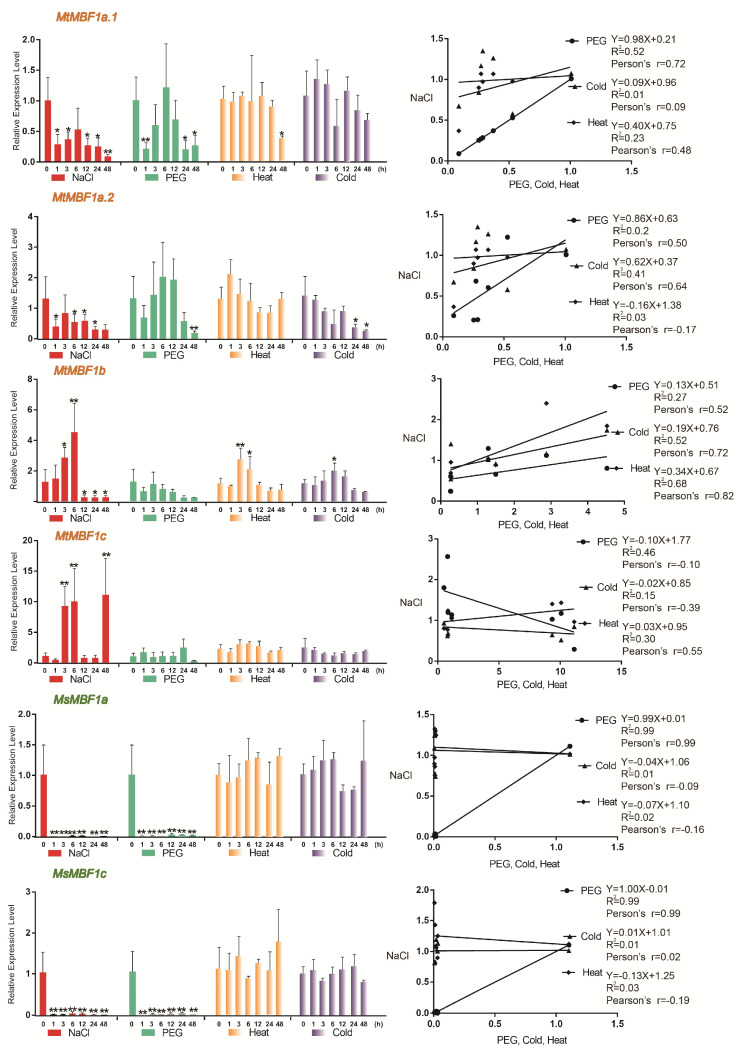
Quantification of the expression levels of the selected *MBF1* genes from *M. truncatula* and *M. sativa* under abiotic stress. Quantification was conducted using qRT-PCR at 0 h, 1 h, 3 h, 6 h, 12 h, 24 h, and 48 h after NaCl, PEG, heat, and cold stress treatments. Expression levels were normalized to the reference gene ACTIN (JQ028730), and RNA was extracted from whole seedlings. Data are the average of three independent biological samples ±SE; the vertical bars indicate standard deviations. ** indicates *p* < 0.01, and * indicates *p* < 0.05. Pearson correlation coefficients (“Pearson”) for expression levels under NaCl, PEG, cold, and heat stresses are shown.

To investigate the response of the *MBF1* genes to various abiotic stress conditions, *Medicago truncatula* and *Medicago sativa* seedlings were treated with 300 mM NaCl, 15% PEG, heat (45 °C), and cold (4 °C) for 0, 1, 3, 6, 12, 24, and 48 h, and the whole seeding were collected for qRT-PCR analysis (Figure 6). The results showed that most *MBF1* genes responded to different environmental stimuli. For instance, under the NaCl, heat (45 °C), and cold (4 °C) treatments, the expression of *MtMBF1b* initially increased, peaked at 3–6 h, and then decreased (Figure 6). *MtMBF1c* exhibited a strong response to NaCl, with expression levels increasing 9-fold, 10-fold, and 12-fold at 3, 6, and 48 h, respectively. Notably, *MsMBF1a* and *MsMBF1c* showed extremely low expression levels in response to both NaCl and drought treatments (Figure 6).

Additionally, we performed a correlation analysis of the expression levels of *Medicago MBF1* genes under the four stress conditions. The results revealed that the Pearson correlation coefficients of *MtMBF1a.1*, *MtMBF1a.2*, *MsMBF1a*, and *MsMBF1c* under the NaCl and PEG treatments were greater than 0.6 (Figure 6). In contrast, when comparing high-temperature and low-temperature treatments with NaCl, only *MtMBF1b* showed a Pearson correlation coefficient greater than 0.6, whereas *MtMBF1c* exhibited a significant negative correlation. These findings suggest that the expression patterns of these genes are similar under NaCl and PEG stresses but differ under high-temperature and low-temperature conditions compared to NaCl stress.

## 3. Discussion

MBF1 is a plant superfamily that has been studied extensively [8]. The gene *AtMBF1* has mainly been studied in the model plant *Arabidopsis*, where it plays a role in the regulation of plant growth, development, and stress responses [13,25]. Following the discovery of four *MBF1* genes in tomatoes by Sanchez Ballesta et al., the MBF1 protein family has been documented in several other plant species [26]. However, a genome-wide identification and characterization of *MBF1* genes has not been undertaken for economically and scientifically valuable plant species of the genus *Medicago*. By conducting an integrated investigation of *MBF1* genes in *Medicago* plants, we identified a total of four *MBF1* members from *M. truncatula* and two *MBF1* members from *M. sativa*.

The results of the multiple sequence matching showed that almost all the *Medicago* MBF1 members contained the MBF1 domains and HTH domains; they also had highly conserved amino acid sequences, a pattern previously documented in tomatoes [26]. We found that *M. truncatula* and *M. sativa* contained at least two *MBF1* genes that were different from those in *Arabidopsis*; these included the key genes *MBF1a* and *MBF1c*. Our phylogenetic analysis showed that *MBF1a* and *MBF1b* were closely related, while *MBF1c* was relatively independent. Interestingly, the MtMBF1c protein was shorter in length than the MtMBF1a and MtMBF1b proteins. However, the *MsMBF1a* gene was significantly shorter in length than *MsMBF1c*, possibly a result of incomplete genome splicing. In addition, the few or absent introns in the gene structure were shown to enhance the plant’s ability to respond to external stresses through rapid transcription and translation. [27].

Gene duplication and differentiation play crucial roles in evolution [28]. We found no evidence for duplication events in both *M. truncatula* and *M. sativa*. However, a collinear relationship between the species was observed (*MtMBF1a.2*-*MsMBF1a* and *MtMBF1c*-*MsMBF1c*). It has been suggested that species displaying a collinear relationship may share similar genetic functions and expression patterns [29]. Hence, the detailed functions and the essential proteins expressed by the *MBF1* genes in *Medicago* species could be elucidated in further studies.

*MBF1* genes play key roles in plant growth and development [30]. By investigating the tissue-specific expression patterns of all the *MBF1* genes in *Medicago*, we found that *MBF1* was mainly expressed in the leaves of these plants. Conditions of high salinity and drought are known to cause the most severe stress for plants [20], while extremes in both heat and cold can also limit their reproduction [31]. There is an urgent need to improve the tolerance of alfalfa to environmental stress to increase the yields of this economically important plant. Subsequently, we verified the expression patterns of all six *MBF1* genes in *M. truncatula* and *M. sativa* under NaCl, PEG, heat, and cold treatments using qRT-PCR analyses. The results revealed distinct expression dynamics between the two species under salt stress (NaCl). Specifically, in *M. truncatula*, *MtMBF1b* and *MtMBF1c* were significantly involved in regulating responses to NaCl stress, with *MtMBF1c* showing robust expression changes—its levels increased 9-fold, 10-fold, and 12-fold at 3, 6, and 48 h, respectively. In contrast, M. sativa *MBF1* genes demonstrated a more subdued response, with *MsMBF1a* and *MsMBF1c* exhibiting extremely low expression levels under NaCl treatment. As a transcription factor, MBF1s mainly function in the nucleus, but it is distributed in both the cytoplasm and the nucleus, and abiotic stress can induce MBF1s to move to the nucleus [31]. The results of this study also showed that MtMBF1a.1, MtMBF1a.2, MtMBF1b, MtMBF1c, and MsMBF1a have obvious nuclear localization, while MsMBF1c only has obvious cytoplasmic localization. MsMBF1c may be induced by abiotic stress before it is localized in the nucleus, which also needs to be verified by subsequent experiments.

In the phylogenetic analysis, we found that the *MBF1* genes were distributed across three branches; the distribution pattern was consistent with *Arabidopsis* and maize [13]. In *Arabidopsis*, the overexpression of *AtMBF1a* has been shown to confer an increased tolerance to infection by the fungal pathogen *Botrytis cinerea* [13]. Likewise, the closely related genes *MtMBF1a.1*, *MtMBF1a.2*, and *MsMBF1a* may be involved in fending off attacks by pathogens. In *Arabidopsis* seedlings, the ectopic expression of *AtMBF1c* did not affect responses to cold stress [14]. In this study, the homologous genes *MtMBF1c* and *MsMBF1c* displayed normal levels of expression under cold stress, suggesting that these two genes are unlikely to play key roles in regulating plant responses to cold stress.

## 4. Conclusions

In this study, we identified six *MBF1* genes from *Medicago sativa* and *Medicago truncatula* on a genome-wide scale. These genes exhibited highly conserved amino acid sequences, motif compositions, and gene structures, suggesting functional similarities. Phylogenetic analysis further revealed the evolutionary relationships between these genes across different plant species, providing crucial insights into their potential roles in response to environmental stresses. Additionally, the expression profiling of the *MBF1* genes in various plant tissues and under different stress conditions highlighted the possible involvement of the *MtMBF1a.2*, *MtMBF1b*, *MsMBF1a*, and *MsMBF1c* gene pairs in regulating NaCl stress responses. While this study provides valuable insights into the evolutionary dynamics and stress-related functions of the MBF1 protein family, further research is needed to experimentally validate the exact molecular mechanisms by which these genes mediate plant stress responses. Future studies could explore the interaction of *MBF1* genes with other transcription factors or signaling pathways, particularly under combined stress conditions such as NaCl. Additionally, functional genomics tools such as CRISPR-Cas9 could be employed to dissect the specific roles of individual *MBF1* genes in stress tolerance, which could ultimately contribute to the development of more resilient crops.

## 5. Materials and Methods

### 5.1. Identification of MBF1 Genes in the M. truncatula and M. sativa Genome

The genomic data of *M. truncatula* and *M. sativa* were downloaded from the databases (https://figshare.com/articles/dataset/Medicago_sativa_genome_and_annotation_files/12623960) (accessed on 12 October 2022) and (https://www.jcvi.org/research/medicago-truncatula-genome-database) (accessed on 12 January 2022). To determine the MBF1 (MBF1 domain (PF08523) and HTH domain (PF01381)) family [13], we first downloaded Hidden Markov Model (HMM) profiles from the Pfam protein family database (https://pfam.xfam.org/) (accessed on 16 October 2022). Next, we obtained the *MBF1* gene sequence of *Arabidopsis thaliana* from the TAIR website [15] (https://www.arabidopsis.org/) (accessed on 17 October 2022). To further screen for *MBF1* genes, we submitted the proposed *MBF1* sequence to InterProScan [13] (https://www.ebi.ac.uk/interpro/search/sequence-search) (accessed on 18 October 2022), CDD (https://www.ncbi.nlm.nih.gov/Structure/bwrpsb/bwrpsb.cgi) (accessed on 18 October 2022), Pfam (https://pfam.xfam.org/) (accessed on 18 October 2022), and SMART (http://smart.embl-heidelberg.de/) (accessed on 18 October 2022). Finally, four *MtMBF1* and two *MsMBF1* genes were identified and located in their corresponding positions on the chromosomes. Molecular weight and pI value are essential for understanding the solubility and stability of transcription factors and their interactions with other proteins and nucleic acids, which are essential for their functional characterization [32]. Subsequently, the molecular weights (MWs) and isoelectric points (pIs) of the deduced amino acid sequences were predicted using the Expert Protein Analysis System (ExPASy) on the proteomics server [13] (https://web.expasy.org/protparam/) (accessed on 20 October 2022). Subcellular localization was predicted using Softberry [15] (http://www.softberry.com/) (accessed on 20 October 2022).

### 5.2. Analyses of Sequences and Structures of the Medicago MBF1 Genes

Identifying conserved motifs using MEME software is crucial to understanding the structural features of MBF1 proteins and predicting their functional mechanisms. To identify conservative motifs in MBF1, we used the MEME software (http://meme-suite.org/tools/meme) (accessed on 25 October 2022), setting the motif number of MBF1 to 10 and the width to a range between 10 and 200 amino acids (aa). Subsequently, we used the jalview software (For version 2.11.4.0) for sequence alignment. We then performed the visualization of the exon/intron positions and the conserved motifs using the TBtools software (For version 0.66) [33].

### 5.3. Phylogenetic Analysis and Classification of MBF1 Genes

The MBF1 proteins of seven plant species (*M. truncatula*, *M. sativa*, *Arabidopsis*, *O. sativa, Z. mays, V. unguiculata*, and *B. rapa*) were used in a multiple alignment in ClustalW [34]. We constructed phylogenetic trees with the neighbor-joining method in the MEGA-X software, with a bootstrap of 1000 replicates. We determined the subfamily clusters for the other species on the phylogenetic tree based on the clustering of *Arabidopsis* species [34]. Subsequently, we used EvolView (https://evolgenius.info/evolview-v2/) (accessed on 1 November 2022) to view the phylogenetic tree.

### 5.4. Analyses of Chromosome Location and Collinearity of MBF1 Genes

The chromosomal locations of all the *MBF1s* of *M. truncatula* and *M. sativa* were obtained from the genome website mentioned above. Multiple collinear scan toolkit (TBtools) was used to analyze gene duplication events with default parameters [35]. All the *MBF1* genes were mapped to eight *M. truncatula* and *M. sativa* chromosomes [33]. To demonstrate intraspecific and interspecific covariance among *M. truncatula*, *M. sativa*, and a representative model plant (*Arabidopsis*), we constructed syntenic maps using the Dual Systeny Plotter software (TBtools for version 0.66). We then used the simple Ka/Ks calculator software to calculate the non-synchronous (Ka) and synchronous (Ks) values of the *MBF1* gene pairs (TBtools) [33].

### 5.5. Subcellular Localization Analysis

For subcellular localization assay, the coding region of *MBF1*s was cloned into the pCAMBIA1300-35S-eGFP vector. This construct was expressed transiently in *Arabidopsis* mesophyll protoplasts using the PEG-mediated method as described by Yoo 2007 [36]. The GFP fluorescence was observed using a confocal microscope (Nikon C2-ER, Tokyo, Japan).

### 5.6. Analyses of Cis-Acting Elements and Locations of MBF1 Genes in Medicago

We used the TBtools software to identify the promoter sequence of the *MBF1* genes (1.5 kb upstream of the translation initiation site), and the program PlantCARE (http://bioinformatics.psb.ugent.be/webtools/plantcare/html/) (accessed on 1 November 2022) to predict *cis*-elements in the promoter region [37]. We then used TBtools to visualize the *cis*-acting elements of all the *MBF1* genes in *Medicago*.

### 5.7. Plant Materials and Treatments

We used plants of the species *M. sativa* (Zhongmu No.1, College of Grassland Science, Qingdao Agricultural University, Qingdao, China) and *M. truncatula* (cv. Jemalong A17, College of Grassland Science, Qingdao Agricultural University, Qingdao, China), which were cultivated at Qingdao Agricultural University. One-month-old *M. truncatula* and *M. sativa* seedlings were used for RNA extraction and qRT-PCR analysis. The plants were grown from seeds in soil under controlled greenhouse conditions with a 16-h light/8-h dark photoperiod at 25 °C. Stress treatments were applied as follows: the soil was irrigated with 200 mL of 300 mM NaCl or 15% mannitol solution to simulate salt and drought stress, respectively; heat stress was induced by placing seedlings in an oven at 45 °C; and cold stress was applied by placing seedlings in a refrigerator at 4 °C. Whole seedlings were collected at 0, 1, 3, 6, 12, 24, and 48 h after each treatment. The samples were immediately frozen in liquid nitrogen and stored at −80 °C for subsequent analysis [38].

### 5.8. Analysis of Gene Expression by qRT-PCR

Total RNAs were extracted using the Eastep^®^ Super total RNA Extraction kit (Promega, Shanghai, China) in accordance with the manufacturer’s instructions. We performed first-strand cDNA synthesis using Trans^®^ Script One-Step gDNA Removal and cDNA Synthesis SuperMix (TransGen Biotech, Beijing, China) in accordance with the manufacturer’s recommendations. We carried out qRT-PCRs using a 2 × RealStar Green Fast Mixture (GeneStar, Shanghai, China) on an ABI 7500 Real-time Detection System [38] (Applied Biosystems, Waltham, MA, USA). We used the housekeeping gene actin-related protein 4A as an internal control. The reaction was prepared as follows: 94 °C for 30 s, followed by 40 cycles of 5 s at 94 °C and 34 s at 60 °C [38]. Finally, we determined the relative expression levels of the different genes using the comparative 2^−ΔΔCt^ method [38]. All the primer sequences used are shown in Table S1.

## Figures and Tables

**Table 1:** Properties of the predicted MBF1 proteins in *M. truncatula* and *M. sativa*.

GeneNumber	Gene Name	TIGR Locus	Start Site	End Site	Homologous Gene	pI	MW (kDa)	Protein Length
MtMBF1-1	*MtMBF1a.1*	MtrunA17_Chr2g0320211	41,480,478	41,484,655	*MsMBF1a*	9.95	15.35	141
MtMBF1-2	*MtMBF1a.2*	MtrunA17_Chr4g0041301	38,998,265	39,003,781	*MsMBF1a*	9.91	15.6	143
MtMBF1-3	*MtMBF1b*	MtrunA17_Chr4g0057871	50,949,726	50,950,391	*MsMBF1a*	8.6	13.75	121
MtMBF1-4	*MtMBF1c*	MtrunA17_Chr6g0485281	39,789,414	39,792,731	*MsMBF1c*	9.9	6.18	56
MsMBF1-1	*MsMBF1a*	MsG0480021982.01.T01	66,798,008	66,798,612	*MtMBF1a.1*	9.45	7.8	74
MsMBF1-2	*MsMBF1c*	MsG0680035636.01.T01	109,794,752	109,795,081	*MtMBF1c*	10.24	11.87	110

## Data Availability

*MtMBF1*: (https://www.jcvi.org/research/medicago-truncatula-genome-database, accessed on 24 November 2024). *Arabidopsis*, *Oryza sativa*, *Zea mays*, *Vigna unguiculata* and *Brassica rapa*: (https://www.uniprot.org/, accessed on 24 November 2024). MBF1 proteins in *M. truncatula* and *M. sativa*: Table 1. Primer information: Table S1.

## Supplementary Materials

The following supporting information can be downloaded at: https://www.mdpi.com/article/10.3390/ijms26020455/s1.
