# Supplementary figures and images for: Genome-wide Characterization of the MBF1 Gene Family and Its Expression Pattern in Different Tissues and Under Stresses in Medicago truncatula and Medicago sativa
Source: Int J Mol Sci. 2025 Jan 8;26(2):455. doi: 10.3390/ijms26020455 (PMC11764565; doi:10.3390/ijms26020455)

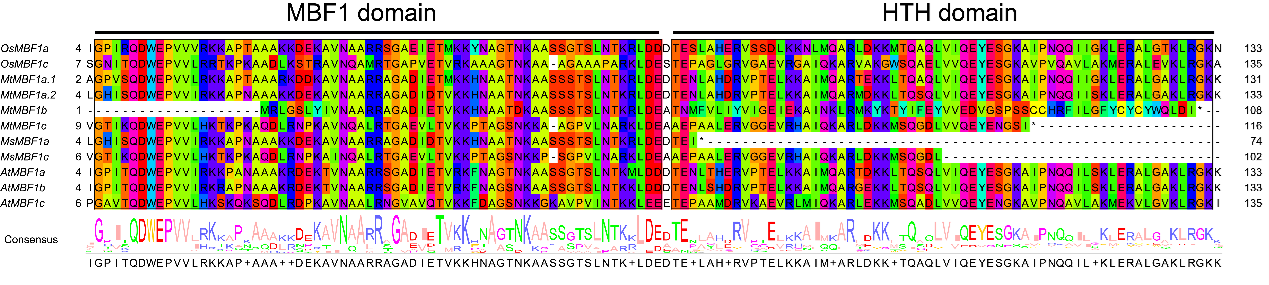

Supplement: Supplementary file 1 [file ijms-26-00455-s001.zip › Figure S1.tif]
